# Transactions of the American and Southern Dental Associations

**Published:** 1889-05

**Authors:** 


TRANSACTIONS OF THE AMERICAN DENTAL ASSOCIATION at the Twenty-eighth Annual Session held at Louisville, Ky., in joint session with the SOUTHERN DENTAL ASSOCIATION. Philadelphia, S. S. White Dental Manufacturing Co , 1889, Publishers.

The volume contains an accurate report of the proceedings of the Louisville meeting, and comprises many valuable papers, some of which are very handsomely illustrated.

The style of the work and its typography are creditable to the publishers.

